# Identification of a Blue Zone in a Typical Chinese Longevity Region

**DOI:** 10.3390/ijerph14060571

**Published:** 2017-05-28

**Authors:** Yi Huang, Geoffrey Mark Jacquez

**Affiliations:** 1 School of Geographic Science, Nantong University, Nantong 226000, China; 2 Department of Geography, State University of New York at Buffalo, Buffalo, NY 14261, USA; gjacquez@buffalo.edu; 3 BioMedware, Ann Arbor, MI 48106, USA

**Keywords:** blue zone, Rugao, degree of aging, GIS, longevity, centenarian

## Abstract

Influenced by a special local environment, the proportion of centenarians is particularly high in some places, known as “blue zones”. Blue zones are mysterious regions that continue to attract research. This paper explores the spatial distribution of the longevity population in a typical Chinese longevity region. Longevity evaluation indexes are used to analyze the longevity phenomenon in 88 towns between 2011 and 2015. Our research findings show that longevity is more important than birth rate and migration in shaping the degree of deep aging in the research region. Fluctuations in the proportion of centenarians are much higher than for nonagenarians, both in relation to towns and to years. This is because there are so few centenarians that data collected over a short time period cannot accurately represent the overall degree of longevity in a small region; data and statistics must be collected over a longer time period to achieve this. GIS analysis revealed a stable longevity zone located in the center of the research region. This area seems to help people live more easily to 90–99 years old; however, its ability to help nonagenarians live to 100 is a weaker effect.

## 1. Introduction

In some regions of the Earth—perhaps influenced by special local soil, water, and air [1,2,3,4,5]—the distribution of the longevity population is geographically clustered, defining what is known as a longevity region (also called a “blue zone” [6]). The proportion of centenarians and people between 90 and 99 years old in the total population are used to define a longevity region; this concept seems easy to understand. Recently, however, as the degree of aging has rapidly increased in some places, the indices used to define a longevity region have become more problematic. A low birth rate decreases the total population, thereby increasing the apparent rate of aging and the proportion of centenarians. For this reason, the proportion of centenarians among nonagenarians (called CI%) and the proportion of nonagenarians in the aging population as a whole (called LI%) have been adopted as new indices when researching the phenomenon of longevity [7]. Statistical data suggest that these two indicators are more useful for measuring the level of longevity in regions with a high degree of aging [8,9,10,11].

**Figure 1:**
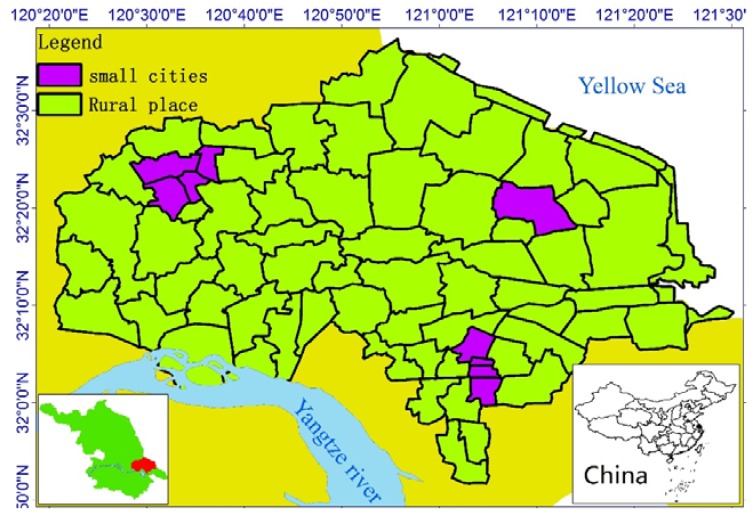
Research region.

The research region of Rugao and the surrounding area on the north shore of the Yangtze River Estuary in China is a famous longevity region [12,13,14]. The ratio of centenarians here is much higher than the average for China or the world. Rugao was voted “China’s longevity region” and “the world’s longevity region” in 2008 and 2011. In addition, the degree of aging in this research area is the highest in China. In 2015, people over 60 years old made up more than 26% of the total population, and was greater than 33% in some towns in this region—about two times higher than the average value in China. According to a population census launched in 2015 by the national bureau of statistics, there are 221.820 million people over 60 years old in China, accounting for 16.15% of the total population [15]. For this reason, the area surrounding Rugao is known as “the oldest country in China”. The reason for the longevity phenomenon and the deep degree of aging here remains unknown; in particular, it is important to determine whether there is any relationship between them. Does longevity account for the fact that there are more old people in this region? What is the spatial–temporal evolution and regulation of longevity here? Is there a core, stable, blue zone in this region? To find answers to these questions, data on aging and longevity were collected in the research area (as shown in Figure 1).

## 2. Materials and Methods

The following 2011–2015 data from a population database owned by the local government (per year per town) were collected:

(1) Total population, population over age 60, population aged 80–89, population aged 90–99, population aged 100 and above in each town. To make the data more accurate, we strictly defined the threshold of each age group: (1) for example, a man/woman must have passed his/her 100th birthday before the data collection date to be classified as a centenarian; (2) the mortality rate for centenarians is higher than 300‰. For this reason, the data should not be counted by year but by day; otherwise, the error will be larger. The cut-off date was set to 31 December. This means that a person who was 90 or 100 years old at the beginning of a year but died before 31 December was not included in the statistics. We found that half of the centenarians died in winter (November to February) in the research region, and the number of centenarians was very small, so the number of centenarians in winter was much lower than summer. Hence, the date must be carefully accounted for to support the accurate calculation of centenarians. We calculated the average number of centenarians on 31 December, 31 March, 31 Jun, and 31 September of a year to represent the number of centenarians of each year. These data were used to calculate the degree of aging, LI% and CI%, and other indices.

(2) Map of each town.

The research region map (Figure 1), ratio maps (Figures 2–7, Figures 10–12), and hot spot analysis (Figures 13 and 14) were generated using ArcGIS 10.2 (Esri, Redlands, CA, USA). The scatter plot (Figures 8 and 9) and univariate local Moran (Figures 15 and 16) were generated using SpaceStat 4.0 (BioMedware, Ann Arbor, MI, USA).

## 3. Results

### 3.1. Value of Each Longevity Indicator

**Figure 2:**
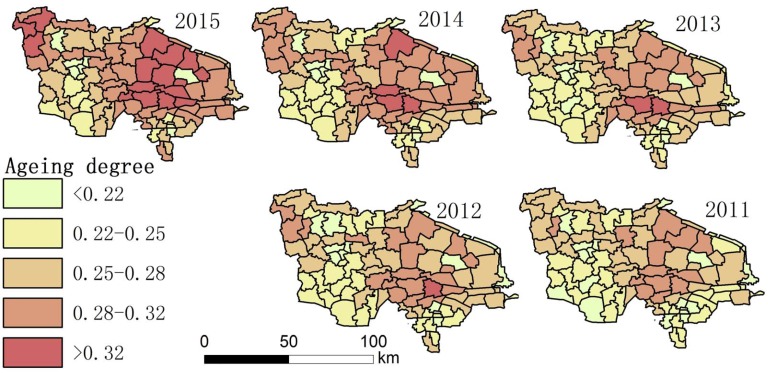
The proportion of the total population over 60 in each town.

**Figure 3:**
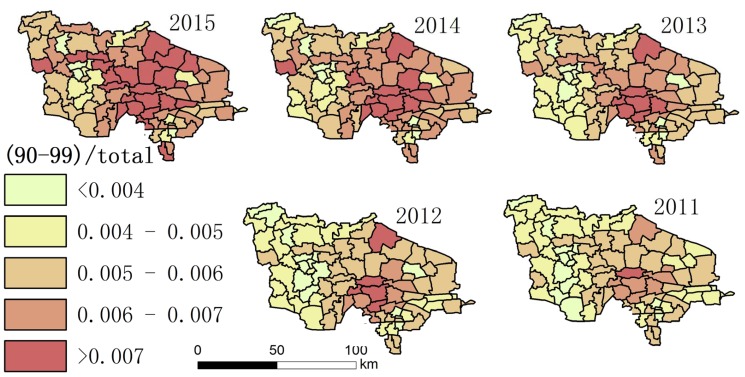
The proportion of the total population aged 90–99 in each town.

**Figure 4:**
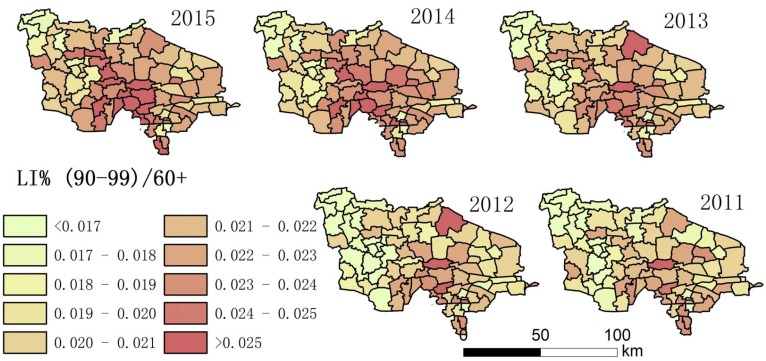
The proportion of people aged 90–99 in the aging population (LI%) of each town.

**Figure 5:**
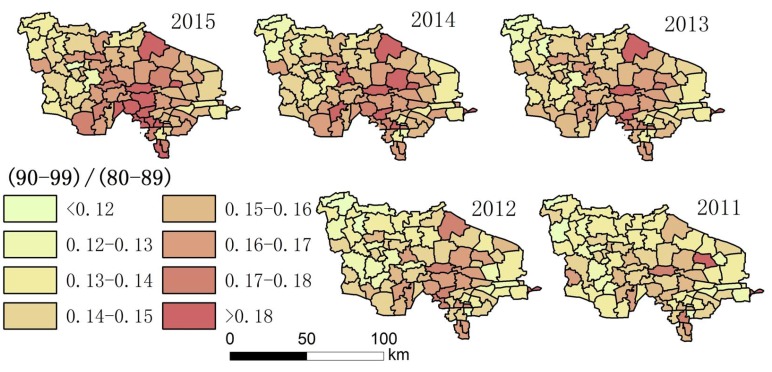
The proportion of the population aged 90–99 in comparison with those aged 80–89 in each town.

**Figure 6:**
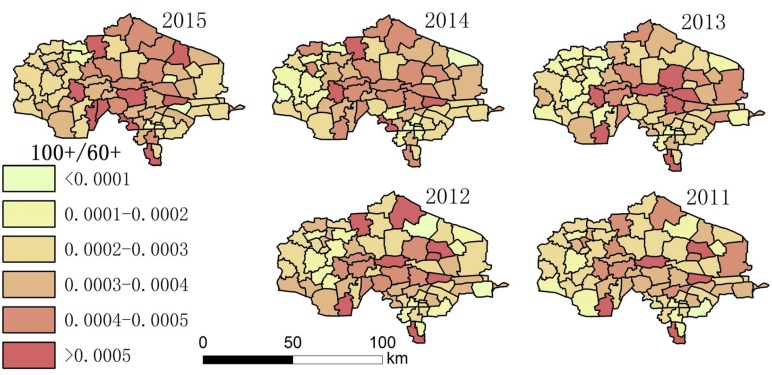
The proportion the aging population of each town made up of people over 100.

**Figure 7:**
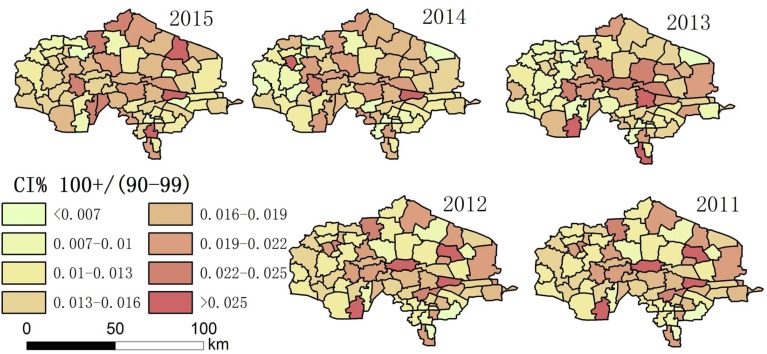
The proportion of the population over 100 in comparison with those aged 90–99 (CI%) in each town.

Using the collected data, the authors drew maps showing the degree of aging and a spatial–temporal map showing longevity character (Figure 2, Figure 3, Figure 4, Figure 5, Figure 6 and Figure 7). For each town in the research area, the classification of intervals represented in the maps used the natural breaks method with equal interval among grades. In order to demonstrate the increasing situation of each ratio from 2011 to 2015, and to make it easier to compare the maps between different years, maps of five years of each ratio were classified by the same interval of value; therefore, maps of each year within a figure share the same legend.

### 3.2. Explanation of the Result

(1) The above data included the entire sample (both male and female), but the male/female ratio was gradually decreasing with increasing age, and there was no obvious difference among towns, as shown in Table 1.

(2) In China, there are two definitions of the ageing society: when the elderly population over 60 years old accounted for 10% of the total population, or 65 years old population accounted for 7% of the total population. The central government prefers to use 65 years old as threshold, while the local government prefers to use 60 years old as threshold, so we chose 60 years old. The degree of aging in the region was the highest in China, increasing year by year. In most areas in 2011, the degree of aging was between 0.22–0.32, with the average degree of aging 0.2408, it reached 0.2774 in 2015, with more than 10 towns exceeding 0.32. Those places with the highest degree of aging are concentrated in the central area. Some areas reached the degree of aging estimated for China in 2050 (0.313) [16].

(3) Figure 4 and Figure 7 show that the distribution of high LI% and CI% was basically stable in 2011–2015 in the study area; the high LI% region was mainly distributed throughout the central area, which is the same distribution pattern in relation to the degree of aging. There was also a high distribution of CI% around this area.

**Figure 8:**
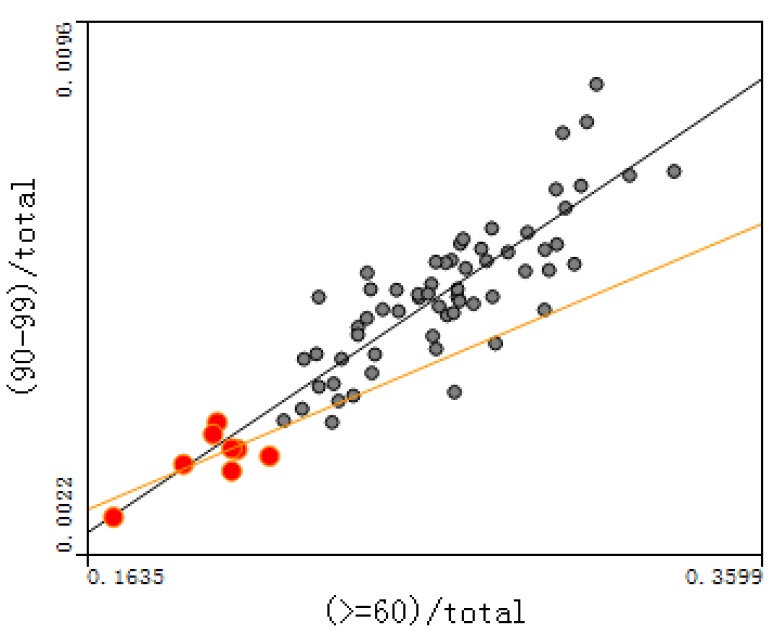
Relationship between the 90–99 ratio and the degree of aging.

**Figure 9:**
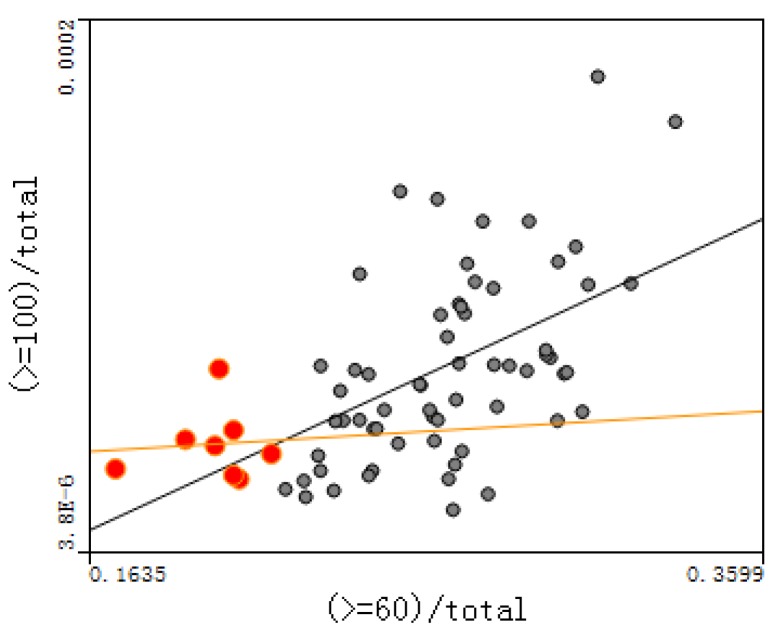
Relationship between the over-100 ratio and the degree of aging.

The relationship between the proportion of the population aged 90–99 and the degree of aging were plotted, and between the proportion of the population aged over 100 and the degree of aging for each town, as shown in Figure 8 and Figure 9 (red circles represent small cities; others rural areas). The correlation coefficients of Figure 8 and Figure 9 are 0.787 and 0.326, respectively; the *p*-values of Figure 8 and Figure 9 are 5.79 × 10^−6^ and 2.28 × 10^−3^, respectively, indicating that the significance is very high.

(4) Figure 8 and Figure 9 show that longevity is deeply associated with the degree of aging, especially for people aged 90–99. This indicates that the longevity phenomenon here decreases the mortality rate, resulting in more old people and leading to a high degree of aging. The relationship between groups aged over 100 and over 60 is also close, but more dispersed than for those aged 90–99. In order to quantitatively analyze the relationship between the degree of aging and each longevity region’s evaluation index, the correlation coefficient between the degree of aging and each index in rural places was calculated, as shown in Table 2.

(4) The residual plot of each regression model was checked; the residual plot shows irregular fluctuation, which means weak autocorrelation. Table 2 shows that the correlation coefficient of the proportion of people with longevity in the aging population is lower than the correlation coefficient of the proportion of people with longevity in the total population. The reason for this is that the high degree of aging has been caused not only by longevity, but also by a low birth rate and the out-migration of young people. The *p*-value of 100/60+ was higher than 0.1 except year 2012, and the correlation coefficient was not as significant as other indicators.

**Figure 10:**
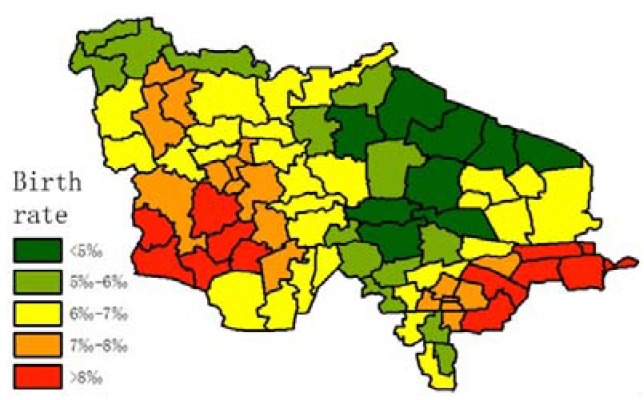
Average birth rate of each town from 2011 to 2015.

**Figure 11:**
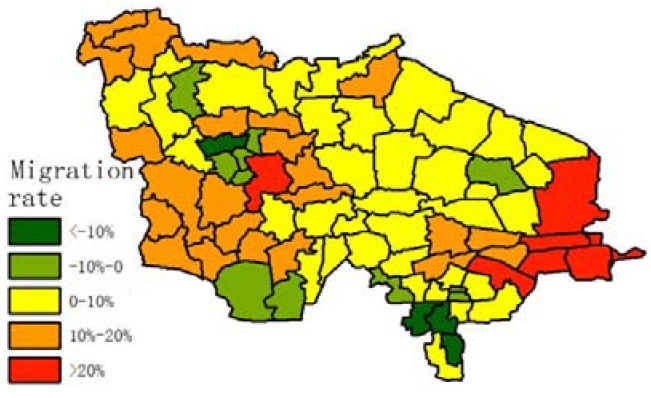
Average migration rate of each town from 2011 to 2015.

Population structure must be considered in order to determine whether longevity is the primary reason leading to a high degree of ageing in the research region. Average birth rate and migration rate data were calculated, as shown in Figure 10 and Figure 11 (the equation of migration rate is as follows):

$$migration\;rate=\frac{\left(Move\;out\;population-Move\;in\;population\right)}{\mathrm{total}\;\mathrm{population}}\times 100$$

In Figure 11, most of the towns are losing population, except for the small cities. The majority of the population who move out of their hometown are young people; they move to big cities out of the research region, and a minority of them move to small cities in the research region.

A multiple liner regression model was built between the degree of ageing (Y), birth rate (X1), migration rate (X2), and longevity (represented by LI%, X3). The R^2^ and F value of the model are 0.5522 and 32.0639, respectively, and parameters X1, X2, and X3 are shown in Table 3.

The significance of the model is very high, as the F value is higher than critical value of F test. Among the parameters, the coefficient of X3 is highest, which means that longevity is the most important factor of high degree of ageing.

We aimed to select the most appropriate index to discover whether there was a stable core longevity zone. Six indices are listed in Table 4, and a variation coefficient is used to evaluate their quality. A high variation coefficient within a year means that the fluctuation of longevity between towns is high, while a high variation coefficient between years means that the index is not stable.

**Figure 12:**
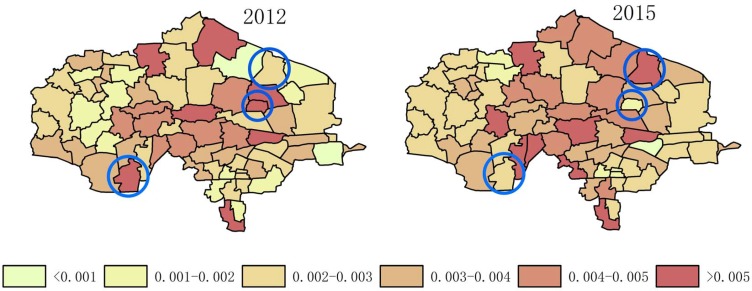
The difference in the proportion of centenarians in the aging population between different years.

Table 4 shows that the average fluctuation of the proportion of centenarians is much higher than that of nonagenarians. This reflects that fact that there are far fewer centenarians than nonagenarians, which creates uncertainty. In Figure 6 and Figure 7, we can see that the centenarian ratio in some towns changed completely from year to year (an enlarged map is shown in Figure 12). When the total population is less than 100,000, the number of centenarians is usually less than 15. As we were preparing the data, we found several people over 100 years old in 2011 (in some towns listed in Figure 6) who were still alive in 2013. In this case, the proportion of centenarians would have been high in the town from 2011 to 2013, but when those individuals died in 2014, there were few nonagenarians to take their place as centenarians (most having died before the age of 99). In this way, the proportion of centenarians dramatically decreased. It is very possible that the ratio will increase again in several years. For this reason, the ratio of centenarians measured over a short period of time cannot represent the degree of longevity in a small region; a longer period of data collection and statistical analysis is essential.

### 3.3. Finding a Stable Blue Zone

**Figure 13:**
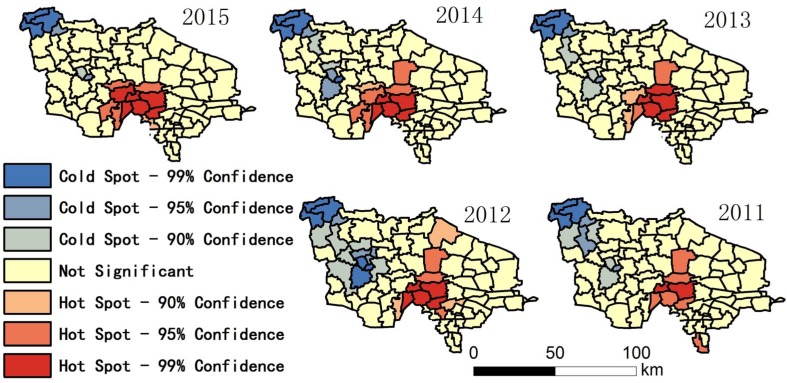
Hot spot in relation to the proportion of people aged 90–99 in the over-60 group.

**Figure 14:**
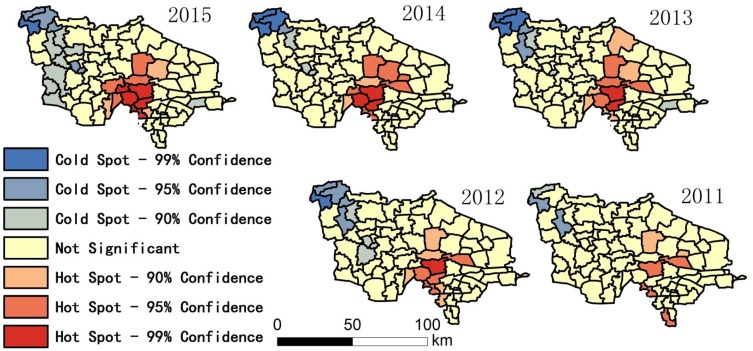
Hot spot in relation to the proportion of people aged 90–99 in comparison with those aged 80–89.

**Figure 15:**
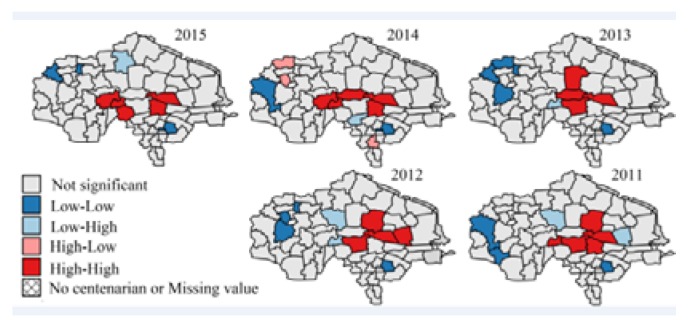
Univariate local Moran: the proportion of people aged over 100 in the over-60 group.

**Figure 16:**
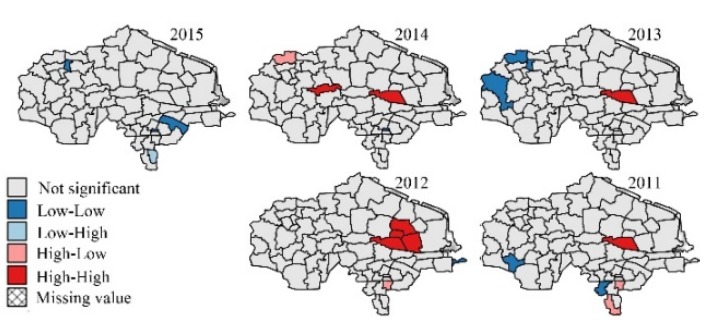
Univariate local Moran: the proportion of people aged over 100 in comparison with those aged 90–99.

Among the indices in Table 1, the fluctuation of the proportion of centenarians and nonagenarians in the total population is higher than that in the aging population and among octogenarians because the birth rate is different in each town, leading to changing population totals. For this reason, the proportion of people with longevity in the total population is not a suitable statistic for evaluation. The indices that look at the proportion of 90–99 year olds in the over-60 group, 90–99 year olds in comparison with those aged 80–89, people over 100 in the over-60 group, and people over 100 in comparison to the 90–99 group were chosen. Hot spot analysis and univariate local Moran were used to find the longevity zone; the hot spot analysis tool identifies spatial clusters of features with high or low values [17]. Univariate local Moran (also called “cluster and outlier analysis”) was used to detect local spatial autocorrelation. It identified local clusters (regions where adjacent areas have similar values) and spatial outliers (areas distinct from their neighbors) [18]. In these two methods, a hot spot or a High–High cluster refers to a town with a high longevity index surrounded by other towns with high longevity indices. A High–Low outlier is a town with a high longevity index surrounded by towns with low longevity indices. A cold spot or Low–Low cluster refers to a town with a low longevity index surrounded by towns that also have low longevity indices. The results of the two methods include Z-scores and *p*-values; the Z-scores and *p*-values tell us whether or not to reject the null hypothesis. For confidence levels of 90, 95, or 99 percent, the corresponding alpha levels are 0.1, 0.05, and 0.01, respectively, and the corresponding Z-scores are 1.65, 1.96, and 2.56, respectively. The results are shown in Figure 13, Figure 14, Figure 15 and Figure 16.

In Figure 13 and Figure 14, hot spot-99%, hot spot-95%, and hot spot-90% means confidence levels of 99, 95, and 90 percent, respectively. In these regions, we can reject the null hypothesis of no spatial clustering in longevity. This is especially true in the hot spot-99% region, which has very high statistical significance.

In Figure 15, the *p*-values in 2011–2015 are all 0.001. In Figure 16, the *p*-values in 2011–2015 are 0.325, 0.002, 0.009, 0.108, and 0.061 respectively, which means the clustering pattern of the proportion of people aged over 100 in the over-60 group is very significant, but the proportion of people aged over 100 in comparison with those aged 90–99 is not significant in 2011 and 2014.

Figure 13, Figure 14, Figure 15 and Figure 16 show that there is a stable longevity zone in the center of the research region (red zone). The proportion of nonagenarians in the aging population and the proportion of nonagenarians to octogenarians is very high here; people easily live to 90–99 years old. However, the proportion of centenarians among nonagenarians is not significantly different from other parts of the research region; red patches are not obvious in Figure 16. The proportion of centenarians in the aging population here is also high, because there are more nonagenarians inside the zone. If the mortality rate of nonagenarians is equivalent, there will certainly be more centenarians here. The longevity zone is located in a rural region, suggesting that a good natural environment and plenty of trace elements in the soil and water make old people in some rural areas live longer, with a better chance of becoming nonagenarians or even centenarians.

## 4. Discussion

By using accurate population data provided by the government, this paper analyzed the spatial distribution of the longevity population in a typical Chinese longevity region. Several indices for blue zone evaluation were used in the study, including those used to measure the number of people aged 90–99 in the total population, those 90–99 in the over-60 population, those 90–99 in comparison with those 80–89, people aged over 100 in the total population, people aged over 100 in the over-60 population, and people over 100 in comparison with those aged 90–99. Linear regression models were used to validate the data, and GIS analysis was used to find blue zones.

## 5. Conclusions

The results of this study show that: (1) The proportion of nonagenarians and centenarians in the total population is influenced by the birth rate, and cannot precisely represent longevity in the region. (2) The paper firstly calculated correlations between birth rate, migration rate, longevity, and high degree of ageing by multiple linear regression model. Analysis showed that longevity is the most important factor that leads to a deep degree of aging in the research region, which is the highest ranked area in China, with a high correlation coefficient between the proportion of people aged 90–99 and the aging ratio. (3) Fluctuations in the proportion of centenarians are much higher than among nonagenarians, both in relation to towns and years; the correlation coefficient and *p*-value of the proportion of centenarians are both not as significant as the proportion of nonagenarians. This is because the number of centenarians is very small. The ratio of centenarians measured over a short period of time cannot represent the degree of longevity in a small region; data gathered over a longer period of time are essential for these statistics. (4) A GIS analysis showed a stable longevity zone located in the center of the research region. This area helps people live more easily to 90–99 years old, but its ability to help nonagenarians live to 100 is weaker (no obvious hot spot in Figure 16). (5) Comparing Figure 2 with Figure 3, Figure 4 and Figure 5, Figure 13, and Figure 14, after the central region, the degree of ageing in the northwest of the research region was ranked second place, but the proportion of longevity population was lowest, which proved the central region was truly a longevity zone. Following on from this research, we plan to sample the soil and vegetation, and to investigate centenarians both inside and outside the core longevity zone to uncover the reason for their longevity.

## Figures and Tables

**Table 1:** Male/female ratio of study region in 2011–2015.

Age	60–64	65–69	70–74	75–79	80–84	85–89	90–94	95–99	100+
Ratio	0.977	1.006	0.957	0.846	0.685	0.5554	0.4404	0.3465	0.1628

**Table 2:** Correlation coefficient (r) and *p* value between the degree of aging and each longevity region evaluation indicator.

Value	90/Total		90/60+		100/Total		100/60+	
	r	*p*-Value	r	*p*-Value	r	*p*-Value	r	*p*-Value
2011	0.6398	0.0238	0.1161	1.29 × 10^−6^	0.3348	0.0010	0.1562	0.2321
2012	0.7212	1.38 × 10^−5^	0.2729	8.49 × 10^−4^	0.3524	1.47 × 10^−4^	0.2208	0.0167
2013	0.7809	2.00 × 10^−6^	0.3238	4.44 × 10^−6^	0.3230	0.0012	0.1617	0.1596
2014	0.7775	6.66 × 10^−6^	0.2983	2.34 × 10^−6^	0.3601	8.11 × 10^−4^	0.1792	0.1872
2015	0.7576	1.86 × 10^−4^	0.2251	2.09 × 10^−8^	0.2768	0.0286	0.0808	0.7553

**Table 3:** Parameter table of multiple liner regression model.

Value	Coefficients	SD	*t* Stat	*p*-Value	Lower 95%	Upper 95%
Intercept	0.3166	0.0242	13.073	0.0000	0.2684	0.3648
X1	−0.8564	0.1005	−8.5176	0.0000	−1.0565	−0.6562
X2	0.0898	0.0160	5.6302	0.0000	0.0581	0.1216
X3	2.6630	0.8654	3.0773	0.0029	0.9402	4.3859

**Table 4:** Fluctuation of each blue zone evaluation index.

Index	100/90	100/60	100/Total	90/80	90/60	90/Total
Average fluctuation within a year	0.39052	0.42142	0.48456	0.11329	0.14175	0.23249
Fluctuation between years	0.09168	0.09482	0.07795	0.02109	0.03665	0.01172
